# PostCOVID-19 Impact on Perinatal Outcomes

**DOI:** 10.3390/diagnostics15010057

**Published:** 2024-12-29

**Authors:** Gaukhar Kurmanova, Ardak Ayazbekov, Almagul Kurmanova, Madina Rakhimbayeva, Gulzhan Trimova, Anarkul Kulembayeva

**Affiliations:** 1 Medicine and Healthcare Faculty, Al Farabi Kazakh National University, 71, Al-Farabi Avenue, 050040 Almaty, Kazakhstan; gaukhar.kurmanova@kaznu.kz (G.K.); trimova@gmail.com (G.T.); 2 Department of Obstetrics and Gynecology, KA Yasawi International Kazakh-Turkish University, 161200 Turkistan, Kazakhstan; 3 City Rheumatology Center, Masanchi Street, 92, 050022 Almaty, Kazakhstan; dr_kulembayeva@mail.ru

**Keywords:** COVID-19, pregnancy, placenta, stillbirth, oligohydramnios

## Abstract

**Background.** Severe Acute Respiratory Syndrome-Related Coronavirus 2 (SARS-CoV-2) infection during pregnancy was associated with a number of adverse pregnancy outcomes, including miscarriage, preeclampsia, preterm birth, and stillbirth. The virus persistence can last for a long time, and the consequences of a previous coronavirus infection are currently under study. **Objectives.** This study aimed to establish the clinical features of the course of pregnancy and childbirth in women with a history of asymptomatic coronavirus disease 2019 (COVID-19). **Methods.** This study was conducted in the Regional Perinatal Center N3 of Turkestan city, Kazakhstan. A total of 229 participants were enrolled comprising individuals with (n = 133, exposed group) from August to September 2021 with a history of COVID-19 and without one in the same period in 2019 (n = 96, unexposed group). **Results.** There is a statistically significant strong association between a history of COVID-19 and the development of oligohydramnios (φ = 0.743, *p* < 0.001), and medium strength between a history of COVID-19 and the presence of anemia (φ = 0.254, *p* < 0.001), abnormal development of the placenta (φ = 0.254, *p* < 0.011), cord entanglement (φ = 0.343, *p* = 0.000), low birth weight (φ = 0.356, *p* < 0.001) and stillbirth (φ = 0.293, *p* < 0.001). **Conclusions.** The past COVID-19 infection in pregnant women has long-term consequences in the form of placenta abnormal development and oligohydramnios; and, as a result, the development of adverse perinatal outcomes

## 1. Introduction

The coronavirus disease 2019 (COVID-19) pandemic has affected all categories of patients, including pregnant women [1,2]. The risk of infection with Severe Acute Respiratory Syndrome-Related Coronavirus 2 (SARS-CoV-2) in pregnant women is 15 times higher than in non-pregnant women, with clinically expressed forms of the disease associated with intoxication, fever, damage to the vascular endothelium, lungs, heart, kidneys, gastrointestinal tract, and the central and peripheral nervous system, leading to maternal and perinatal mortality and morbidity [3]. Pregnancy is an independent risk factor for adverse outcomes in women with SARS-CoV-2. Pregnancy alters the immune system’s response to viral and bacterial infections in general, including COVID-19, and leads to the development of severe clinical symptoms. The impact of SARS-CoV-2 infection on the course and outcomes of pregnancy is unfavorable [4]. Thus, it was shown that the perinatal mortality rate was about 4%, and the main factors determining adverse perinatal outcomes with maternal COVID-19 infection are early gestational age, the presence of mechanical ventilation in the mother, and low birth weight [5]. Pregnant women with SARS-CoV-2 infection had significantly higher risk of developing preeclampsia compared to women without infection (pooled odds ratio [OR] 1.62; 95% confidence interval [CI] 1.45–1.82), with both asymptomatic and symptomatic SARS-CoV-2 infection significantly increasing the risk of preeclampsia [6]. A meta-analysis showed that SARS-CoV-2 infection was associated with preterm birth, stillbirth, and lower birth weight compared to no SARS-CoV-2 infection [7]. In a large population-based study, a COVID-19 diagnosis increased the risk of very-preterm (<32 wk), preterm (<37 wk), and early (37 and 38 wk) birth, especially among people with comorbidities [8]. An increased risk of stillbirth has been associated with virus infection when pregnant individuals were infected during early (<20 wk) and mid-pregnancy (21–27 wk), suggesting potential fetal vulnerability to SARS-CoV-2 infection in early pregnancy [9].

Angiotensin-converting enzyme-2 and transmembrane serine protease 2, which play an active role in the entry of the SARS-CoV-2 into the cell, are also co-expressed in the placenta, indicating that the placenta is an organ at increased risk for COVID-19 [10].

This creates conditions for the penetration of the coronavirus into the placenta cells and the subsequent development of placental dysfunction, including feto–maternal vascular malperfusion [11,12]. There are also reports of the development of severe oligo- and anhydramnios in SARS-CoV-2-positive pregnant women [13,14,15].

All consequences of COVID-19 are associated with complex pathological changes caused by the effect of the SARS-CoV-2 coronavirus on the human body. There are increasing reports of a cohort of women with an asymptomatic or mildly symptomatic infection, which in most cases does not attract attention (mild to moderate cold/flu-like symptoms). The course and outcome of pregnancy in clinically silent forms of COVID-19 (mild form) and in individuals with an asymptomatic history (post-COVID-19) remain poorly understood. The seroprevalence of SARS-CoV-2 in pregnant women at the time of delivery in the period April–June 2020 was 4.5–14%; however, the extent and duration of protection by SARS-CoV-2 IgG antibodies remain unknown [16]. The seroprevalence of SARS-CoV-2 in women was 50% in the Republic of Kazakhstan in June 2021 [17].

The virus persistence can last for a long time, and the consequences of a previous (including asymptomatic) coronavirus infection (post-COVID) are currently under study [18,19]. The findings from an integrative review indicated that four pathophysiological categories are involved in post-COVID syndrome: virus-specific pathophysiological variations, oxidative stress, immunologic abnormalities, and inflammatory damage [20,21]. Therefore, the purpose of this study was to determine the impact of a past COVID infection on the course and outcomes of pregnancy.

## 2. Materials and Methods

### 2.1. Data Collection

This study was conducted in the Regional Perinatal Center N3 of Turkestan, Kazakhstan. Data were collected from medical records of 229 participants aged 18 to 42 years admitted to hospital for delivery in the period August–September 2021 with seropositive antibodies to SARS-CoV-2 IgG (n = 133, exposed cohort), compared with patients in the same period in 2019 (n = 96, pre-pandemic cohort, respectively, not exposed to coronavirus, therefore no antibodies and not vaccinated against coronavirus).

Pregnant women hospitalized for delivery in August to September 2021 and suspected of being infected with coronavirus (having contact with patients with coronavirus infection) were examined for the presence of antibodies against the coronavirus. The serum samples were examined for SARS-CoV-2 antibodies detection using enzyme-linked immunosorbent assay (ELISA), Vector-Best, Novosibirsk, Russian Federation). The ELISA detects the antibodies class G immunoglobulins (IgG) and class M (IgM) to the SARS-CoV-2 receptor-binding domain of spike glycoprotein (S-protein). The IgG antibody test shows the level of immune response and indicates recent or past exposure to the virus (including subclinical or asymptomatic infection).

The exposed cohort had the following criteria:

Inclusion criteria: pregnant women hospitalized for delivery, over the age 18, unvaccinated against COVID-19, had SARS-CoV-2 IgG seropositive antibodies.

Exclusion criteria: comorbidity (kidney and blood diseases, systemic autoimmune diseases, thyroid disorders, malignancies, chronic inflammatory disease, tuberculosis, diabetes mellitus); the presence of IgG antibodies against Herpes simplex virus, Cytomegalovirus, Toxoplasma gondii, Chlamydia trachomatis, Ureaplasma urealyticum; and vaccinated against coronavirus.

The unexposed group had the following criteria:

Inclusion criteria: pregnant women hospitalized for delivery, over the age 18, without antibodies against coronavirus and unvaccinated against COVID-19.

Exclusion criteria: comorbidity (kidney and blood diseases, systemic autoimmune diseases, thyroid disorders, malignancies, chronic inflammatory disease, tuberculosis, diabetes mellitus); and the presence of IgG antibodies against Herpes simplex virus, Cytomegalovirus, Toxoplasma gondii, Chlamydia trachomatis, and Ureaplasma urealyticum.

Procedures and Laboratory Measurements: a structured checklist tool was developed before the actual data collection. Data were collected from the medical records of participants. The demographic information, including age, body mass index (BMI), parity, current pregnancy, laboratory parameters (general blood count, urinalysis, C-reactive protein), ultrasound examination of the placenta and fetal membranes, birth outcomes (vaginal birth or cesarean section, emergency or planned births), perinatal outcomes (Apgar score at 1 and 5 min, fetal weight) were extracted from their records. According to the WHO classification, an adult BMI value below 18.5 was considered underweight, from 18.5 to 24.9 was considered healthy weight, from 25 to 29.9 as overweight, and 30 and above was considered obese.

Pregnancy hypertension was diagnosed when systolic blood pressure was above 140 mm Hg and/or diastolic blood pressure >90 mm Hg. Reference intervals for the main parameters of the clinical blood test are as follows: mild anemia—Hb 100–109 g/L; moderate—Hb 70–99 g/L; severe—Hb below 70 g/L. Leukopenia was detected at values below 4 × 10^9^/L, and leukocytosis above 10 × 10^9^/L. Trombocytopenia was detected at values <150 × 10^6^/L and trombocytosis >400 × 10^6^/l. Proteinuria was considered in the presence of protein in the urine ≥0.3 g/day or ≥0.3 g/L in a double urine test.

The amniotic fluid volume can be assessed by ultrasound device Voluson E8. The most commonly used ultrasound criteria for oligohydramnios are a single deepest pocket <2 cm and an amniotic fluid index <5 cm, and for anhydramnios an absence of amniotic fluid [22]. To date, the main echographic sign and criterion for oligohydramnios is a decrease in the amniotic fluid index (AFI), which is calculated as the sum of the vertical dimensions of the maximum pockets of amniotic fluid, defined in four quadrants. Oligohydramnios is a condition with an AFI <10 cm (for up to 34 wk) or <8 cm (for >34 wk). Moderate oligohydramnios—AFI > 5 cm, critical oligohydramnios—AFI < 5 cm. Intrauterine growth restriction (IUGR) is diagnosed when the fetal weight estimated by ultrasound is below the 10th percentile for gestational age with deranged Doppler parameters [23].

Sample Size. The sample size for the unmatched cohort study was determined using “Epi infoTM” (Epi Info™|CDC, accessed on 28 December 2024) via the following methodology: two-sided confidence level—95%; power—80%, ratio (unexposed: exposed)—0.5. Percent outcome in the unexposed group was 5%, risk ratio was 4; odds ratio was 4.75. Percent outcome in the exposed group was 20%. The preliminary calculation resulted in the sample size of n = 201 (134/67). The percentage of errors α—5% and β—20%, and samples loss—10%. Since the predicted response is 90%, the sample will need to be increased by 10%, and the sample size will be 201 × 110% ≈ 211 people.

### 2.2. Ethics Approval

This study was approved by the Ethical Committee of Al Farabi Kazakh National University, Kazakhstan (Code: IRBA492/IRB 00010790). Only clinical data collected as part of routine clinical care were used in this project, and therefore informed consent was not obtained.

### 2.3. Statistical Analysis

Statistical analysis was carried out using IBM Statistical Package for the Social Sciences, version 26, SPSS Inc., Armonk, NY, USA. All quantitative indicators were coded and converted to nominal. Categorical variables were shown as absolute values (n) and percentages (%). A Chi-square test or Fisher’s exact test were performed for the variables. A comparison of nominal data was carried out using the Chi-square test. If the obtained value exceeded the critical value, it was concluded that there was a statistical relationship between the studied risk factor and the outcome at the appropriate level of significance. In the case of the analysis of four-field tables with the expected phenomenon in at least one cell less than 10, we calculated the Chi-square with the Yates correction, which makes it possible to reduce the probability of an error of the first type, i.e., detecting differences where there are none. The statistical significance was set at *p* < 0.05.

The strength of the connection was estimated by the criterion φ. Interpretation of the obtained values of statistical criteria according to the recommendations of Rea and Parker: < 0.1—insignificant, 0.1–0.2—weak, 0.2–0.4—medium, 0.4–0.6—relatively strong, 0.6–0.8 —strong, and 0.8–1.0—very strong. The OR and 95% CI were used to estimate the associations between the underlying factors and perinatal outcomes. Statistical significance was defined as *p* < 0.05.

## 3. Results

The clinical characteristics of the exposed and unexposed groups are presented in Table 1.

No differences in age, body mass index or parity of delivery were found when comparing the participants in both groups. The current pregnancy was complicated in both groups. The presence of hypertension (without proteinuria or preeclampsia) was approximately the same in the exposed and unexposed groups. It should be noted that mild anemia was seen more often in the unexposed group (*p* = 0.038). No statistically significant differences were found in the examination of laboratory parameters. However, thrombocytopenia was more frequent in the unexposed group (*p* = 0.019).

Table 2 shows the perinatal outcomes.

There were no differences in terms (*p* > 0.05) and methods of delivery (*p* > 0.05) between groups. In the exposed group, the weight of newborns < 2500 g (*p* < 0.01), entanglement of the umbilical cord (*p* < 0.01), and intrauterine growth restrictions (*p* = 0.006) were significantly more frequent than in the unexposed group. Differences were also found in scores on the Apgar scale; low scores (up to 3) and average scores (4–6) were recorded significantly more frequently in the exposed group than in the unexposed group after 1 min (*p* = 0.022 and *p* < 0.001, respectively) and average scores (4–6) after 5 min (*p* = 0.014).

Table 3 shows the condition of the placenta and amniotic fluid in the exposed group and the unexposed groups.

Abnormalities in the development of the placenta were significantly more frequent in the exposed group than in the unexposed group (*p* = 0.005). The early maturation of the placenta was more often recorded in the exposed group, compared to the unexposed group (*p* = 0.014).

In our study, one of the most important signs of the consequences of coronavirus infection deserves special attention—in the exposed group, oligohydramnios (86.4% versus 11.5%, *p* < 0.01) and absolute oligohydramnios (ahydroamnios) (9.0% versus 0%) were observed significantly more often, compared to the unexposed group (*p* = 0.003).

The next step was to study the correlation between the presence of a history of COVID-19 and perinatal outcomes (Table 4).

A weak association was found between a history of COVID-19 and intrauterine growth restriction (φ = 0.184, *p* = 0.005, OR = 1.803 95%, CI (1.602–2.030). There is a statistically significant association of medium strength between a history of COVID-19 and the presence of anemia (φ = 0.254, *p* < 0.001), abnormal development of the placenta (φ = 0.254, *p* < 0.011), cord entanglement (φ = 0.343, *p* < 0.001, OR = 10.97 95% CI (3.78–31.83), low birth weight (φ = 0.356, *p* < 0.001), OR = 35.225 95% CI (4.718–262.983) and stillbirth (φ = 0.293, *p* < 0.001). Correlation analysis revealed a statistically significant strong correlation between a history of COVID-19 and the development of oligohydramnios (φ = 0.743, *p* < 0.001, OR = 46.995 95% CI (21.463–102.899).

## 4. Discussion

Since the World Health Organization (WHO) declared the COVID-19 pandemic, the entire world has been facing an unprecedented global crisis. Kazakhstan was affected by the wave caused by the Delta variant of COVID-19, which began in June and peaked in mid-August 2021 [17]. All categories of patients were affected by the COVID-19 pandemic, including pregnant women. SARS-CoV-2 infection during pregnancy has been associated with a range of adverse pregnancy outcomes, such as early pregnancy loss, preterm delivery, preeclampsia, fetal death, vertical transmission, intrauterine growth restriction, and congenital structural anomalies. COVID-19 is characterized by multisystemic damage to the body, the severity of which varies from asymptomatic carriage to death.

A significant proportion of COVID-19 cases are asymptomatic, and this large group of asymptomatic cases may include a number of people who have tested positive for SARS-CoV-2 IgG, including pregnant women.

The effects of the coronavirus on organs and systems last a long time after infection. Between 2% and 13% of people suffer long-term symptoms after COVID-19 infection, and there is a risk of developing this condition during pregnancy. Identifying the association between adverse fetal outcomes and SARS-CoV-2 during pregnancy is critical for prognosis, early intervention, and prevention of complications.

In our study, a retrospective analysis of the birth histories of 133 patients with a history of COVID-19 (main group) in the period August–September 2021 compared to 96 patients in the same period in 2019 (before the COVID-19 pandemic) was performed. The first cohort were pregnant women who, during the pandemic, had the SARS-CoV-2 IgG seropositive antibody, while they did not receive vaccinations. The consequences of COVID-19 infection were observed in SARS-CoV-2 IgG seropositive pregnant women: a significant increase in the incidence of low-birthweight newborns (24.8% versus 1.04%, *p* < 0.01) with intrauterine growth restriction (7.5% versus 0%), and placenta abnormalities (17.3% versus 4.2%, *p* = 0.005) with the development of oligohydramnios. One of the most important signs of the consequences of coronavirus infection deserves special attention—the development of oligohydramnios (86.4% versus 11.5%, *p* < 0.01) and absolute oligohydramnios (ahydroamnios) (9.0% versus 0%). A statistically significant moderate-strength association was found between a history of COVID-19 and anemia, placental abnormalities, low birth weight, and stillbirth (φ = 0.293, *p* ≤ 0.001); and a significant strong association was found for the development of oligohydramnios (φ = 0.743, *p* = 0.000).

The research on pregnant women with COVID-19 infection results in SARS-CoV-2 placentitis. SARS-CoV-2 placentitis, which is manifested as increased fibrin deposition, trophoblast necrosis, and intervillositis, prevents blood perfusion in the intervillous space, leading to the destruction of the placental parenchyma [24]. A study of pregnancy outcomes in patients with COVID-19 showed an increased risk of stillbirth compared to uninfected women, especially during the Delta’s variant predominance [14]. Stillbirths (0.7% vs. 0.4%; adjusted prevalence ratio 1.55; 95% confidence interval 1.14–2.09) and preterm births (12.8% vs. 11.9%; adjusted prevalence ratio 1.14; 95% confidence interval 1.07–1.20) were recorded more frequently during the period in which the Delta variant of the virus was circulating, compared to the period before the Delta variant [25].

Women infected with coronavirus, who have not been vaccinated, have a much higher risk of stillbirth or death of their child in the first month of life than pregnant women in general [26].

However, our results are comparable to a case-control study, which reported an increase in the umbilical-artery and uterine-artery resistance index in pregnant women with a history of COVID-19 at third week compared to women who had no history of exposure to SARS-CoV-2 [27], as well as with another study that found a case of intrauterine growth restriction associated with severe oligohydramnios and loss of fetal movement in a term pregnancy with a history of positive SARS-CoV-2 at 32 weeks’ gestation [14]. In contrast, no difference in amniotic fluid index was found between women who tested positive for SARS-CoV-2 and uninfected pregnant women, when analyzing ultrasound results [28]. However, there is persistence of the virus with long-term effects leading to placental insufficiency, which ultimately results in damage to the organs of the fetus, leading to extensive intervillous deposition of fibrinoids with subsequent formation of placental infarction and ischemia [29]. Pregnant women infected with COVID-19 have a higher incidence of obstetric complications, including a 25% increased risk of premature birth and intrauterine growth restriction [29].

Highly immunogenic viral infections such as SARS-CoV-2 can interrupt the normal course of pregnancy. COVID-19 causes uncontrolled systemic inflammation. In pregnant women infected with SARS-CoV-2, an imbalance between Treg and Th17 cells may be associated with adverse pregnancy outcomes such as pregnancy loss, preterm delivery and preeclampsia [30]. In this regard, different results are possible, depending on individual immune susceptibility and gestational age at the time of onset of infection, which can bidirectionally determine both obstetric complications and the duration and/or severity of the infectious disease [31].

It has been established that not only does the active form of the coronavirus infection have a significant negative impact on the human body, but it also plays an important role in long-term (more than 12 weeks) post-COVID syndrome, which is characterized by both general clinical manifestations and damage to the vascular wall and endothelium, a tendency to hypercoagulation and microthrombosis. Coronavirus infection suffered before pregnancy contributes to a 52.4% and 86.9% increase in the incidence of pre-eclampsia and placental insufficiency in the second trimester and a two-fold increase in the incidence of placental insufficiency and fetal hypoxia in the third trimester, compared to healthy women [32]. It was also found that patients with a history of COVID-19 had various symptoms of thrombohemorrhagic syndrome [33]. These symptoms are caused by endothelial cell dysfunction induced both directly by the virus and by the developing cytokine storm, and subsequently by autoimmune damage [34,35]. Further research documents immunological responses at the maternal–fetal interface, placental disfunctions caused by SARS-CoV-2 infection and routes of entry into placental cells. The study also notes the genomic and proteomic features of SARS-CoV-2 studied to date, as new variants of the virus appear that are characterized by increased transmissibility and more efficient immune-escape techniques [36]. Another study reports that infection with SARS-CoV-2 during pregnancy increases the possibility of an unfavorable outcome. According to histopathological studies, SARS-CoV-2 affected the placenta as a maternal–fetal interface and led to inflammation and systemic vasculopathy. More specifically, 66.7% of the placentas in the case group had histopathological abnormalities characterized as vascular or inflammatory abnormalities in the pregnant women diagnosed with SARS-CoV-2. Acute ischemia–infarct areas were observed in 22.2% of placentas. The percentage of placentas with subchorionic layer thrombi was 8.3% [37].

The history of COVID-19 increases the risk of abnormal placental development and oligohydramnios, and thus the development of adverse perinatal outcomes with low birthweight; these require more careful monitoring of pregnancy.

A limitation of this study was in the impossibility of testing the placenta for the presence of SARS-CoV-2 by polymerase chain reaction. In this case, there would be direct evidence of the influence of the transferred asymptomatic form of COVID-19 on the development of placental insufficiency.

## 5. Conclusions

This study examined the course of pregnancy in patients with a history of asymptomatic COVID-19. According to the results of this study, there were no significant differences between groups in assessing risk factors (age, BMI), the course of pregnancy, the presence of hypertensive conditions (preeclampsia), the timing and outcomes of labor (vaginal delivery or cesarean section), or laboratory parameters. However, differences were found in parameters such as placental abnormalities, low birth weight, intrauterine growth restriction, cord entanglement, and oligohydramnios (including anhydramnios).

## Figures and Tables

**Table 1:** Demographic and clinical characteristics of groups.

Variables	Exposed (n = 133)	Unexposed (n = 96)	*p*-Value
Variables	Exposed (n = 133)	Unexposed (n = 96)	*p*-Value
Age at delivery (yrs):			
18–28	60 (45.1)	49 (51.0)	0.545
>29	73 (54.9)	47 (49.0)	0.560
BMI:			
18.5–24.9 (reference weight)	39 (29.3)	38 (39.6)	0.216
25–30 (overweight)	62 (46.6)	38 (39.6)	0.448
>30 (obesity)	32 (24.1)	20 (20.8)	0.630
Parity:			
Nulliparous	43 (32.3)	27 (28.1)	0.588
Multiparous	90 (67.7)	69 (71.9)	0.721
Hb (g/L):			
>109	57 (42.8)	16 (16.7)	0.001
100–109 (mild anemia)	55 (41.4)	60 (62.5)	0.038
70–99 (moderate anemia)	21 (15.8)	20 (20.8)	0.408
Tension in pregnancy:			
Normotension	98 (73.7)	79 (82.3)	0.435
Hypertension without proteinuria	23 (17.3)	9 (9.4)	0.125
Preeclampsia	12 (9.0)	8 (8.3)	0.886
Leukocytes:			
Leukocytosis	62 (46.6)	42 (43.8)	0.763
Leukopenia	4 (3.0)	1 (1.0)	0.067
Thrombocytes:			
Thrombocytopenia	5 (3.4)	13 (13.5)	0.019
Thrombocytosis	16 (12.0)	5 (5.2)	0.100
CRP (mg/L):			
<5	111 (83.5)	86 (89.6)	0.642
>6	22 (16.5)	10 (10.4)	0.238
Proteinuria	29 (21.8)	12 (12.5)	0.112

Note. BMI: Body mass index, Hb: Hemoglobin, CRP: C-reactive protein.

**Table 2:** Perinatal outcomes.

Variables		Exposed (n = 133)	Unexposed (n = 96)	*p*-Value
Gestation period				
	Term (38 wk)	104 (78.2)	69 (71.9)	0.448
	Premature (<38 wk)	27 (20.3)	27 (28.1)	0.330
	Overdue (>41 wk)	2 (1.5)	0 (0)	0.220
Birth outcome				
	Vaginal delivery	85 (63.9)	70 (72.9)	0.442
	Cesarean section	48 (36.1)	26 (27.1)	0.258
Fetus				
	Weight of the child (over 2500 g)	91 (68.4)	93 (96.9)	0.027
	Weight of the child (up to 2500 g)	33 (24.8)	1 (1.0)	<0.01
	Stillbirth	9 (6.8)	2 (2.1)	0.115
Entanglement of the umbilical cord		43 (32.3)	4 (4.2)	<0.01
Intrauterine growth restriction		10 (7.5)	0	0.006
Apgar for 1 min				
	0–3	16 (12.0)	3 (3.13)	0.022
	4–6	21 (15.8)	1 (1.04)	<0.001
	7–10	96 (72.2)	92 (95.83)	0.068
Apgar for 5 min				
	0–3	13 (9.8)	3 (3.1)	0.06
	4–6	8 (6.0)	0	0.014
	7–10	112 (84.2)	93 (96.9)	0.34

Note. Data presented as n (%). Chi-square test.

**Table 3:** The state of the placenta and amniotic fluid.

Variables		Exposed (n = 133)	Unexposed (n = 96)	*p*-Value
Abnormalities of the placenta:		23 (17.3)	4 (4.17)	0.005
	Early maturation	8 (6.02)	0	0.014
	Placentitis	7 (5.26)	1 (1.04)	0.092
	Placenta previa	3 (2.26)	0	0.133
	Placenta accreta	4 (3.01)	0	0.08
	Placental abruption	1 (0.75)	3 (3.13)	0.22
Oligohydramnios:		115 (86.4)	11 (11.5)	<0.001
	Moderate	103 (77.4)	11 (11.5)	<0.001
	Critical (anhydramnios)	12 (9.0)	0	0.003

Note. Data presented as n (%). Chi-square test.

**Table 4:** Associations between a history of COVID-19 and pregnancy outcomes.

Variables	φ	*p*-Value	OR (95%CI)
Anemia	0.254	<0.001	-
Proteinuria	0.128	0.053	2.038 (0.981–4.234)
Anomalies of the placenta	0.254	0.011	-
Entanglement of the umbilical cord	0.343	<0.001	10.967 (3.71–31.83)
Intrauterine growth restriction	0.184	0.005	1.803 (1.602–2.030)
Low birth weight	0.356	<0.001	35.225 (4.718–262.983)
Stillbirth	0.293	<0.001	-
Oligohydramnios	0.743	<0.001	46.995 (21.463–102.899)

Note. φ: strength of the connection, OR: odds ratio, CI: confidence interval.

## Data Availability

The data presented in this study are available on request from the corresponding author. The data are not publicly available due to privacy and ethical issues.
